# Dataset of oxygen, carbon, and strontium isotope values from the Imperial Roman site of Velia (ca. 1st-2nd c. CE), Italy

**DOI:** 10.1016/j.dib.2021.107421

**Published:** 2021-09-28

**Authors:** Robert J. Stark, Matthew V. Emery, Henry Schwarcz, Alessandra Sperduti, Luca Bondioli, Oliver E. Craig, Tracy L. Prowse

**Affiliations:** a Department of Anthropology, McMaster University, Chester New Hall Rm. 524, 1280 Main Street West, Hamilton, Ontario L8S 4L9, Canada; b School of Human Evolution and Social Change, Arizona State University, 900 Cady Mall, Tempe, AZ 85281, USA; c School of Geography and Earth Sciences, McMaster University, General Science Building Rm. 302, 1280 Main Street West, Hamilton, Ontario L8S 4L9, Canada; d Dipartimento Asia Africa e Mediterraneo, Università degli Studi di Napoli “L'Orientale”, Piazza S. Domenico Maggiore, 12, Napoli 80134, Italy; e Servizio di Bioarcheologia, Museo delle Civiltà, Piazza G. Marconi 14, Rome 00144, Italy; f Department of Archaeology, BioArCh, University of York, Environment Building, Wentworth Way, Heslington, York YO10 5DD, United Kingdom

**Keywords:** Southwestern Italy (Campania), Cilento of Lucania, Isotope analyses, Dental enamel, Imperial Roman, Human mobility, Fourier transform infrared spectroscopy (FTIR), Crystallinity index (CI)

## Abstract

The oxygen (δ^18^O_carbonate_), strontium (^87^Sr/^86^Sr), and previously unpublished carbon (δ^13^C_carbonate_) isotope data presented herein from the Imperial Roman site of Velia (ca. 1st to 2nd c. CE) were obtained from the dental enamel of human permanent second molars (M2). In total, the permanent M2s of 20 individuals (10 male and 10 female) were sampled at the Museo delle Civiltà in Rome (formerly the Museo Nazionale Preistorico Etnografico “L. Pigorini”) and were subsequently processed and analysed at McMaster University. A subsample of teeth (n=5) was initially subjected to Fourier transform infrared spectroscopy (FTIR) analysis to assess for diagenetic alteration through calculation of crystallinity index (CI) values. Subsequently, tooth enamel was analysed for δ^13^C_carbonate_ and δ^18^O_carbonate_ (VPDB) using a VG OPTIMA Isocarb isotope ratio mass spectrometer (IRMS) at McMaster Research for Stable Isotopologues (MRSI), and ^87^Sr/^86^Sr was measured by dynamic multi-collection using a thermal ionization mass spectrometer (TIMS) in the School of Geography and Earth Sciences. The dental enamel isotope data presented represent the first δ^18^O, δ^13^C_carbonate_, and ^87^Sr/^86^Sr values analysed from Imperial Roman Campania to date, providing data of use for comparative analyses of δ^18^O, δ^13^C, and ^87^Sr/^86^Sr values within the region and for assisting in documenting human mobility in archaeological contexts. Full interpretation of the δ^18^O and ^87^Sr/^86^Sr data presented here is provided in “Imperial Roman mobility and migration at Velia (1^st^ to 2^nd^ c. CE) in southern Italy” [1].

## Specifications Table

**Table:** 

Subject	Archaeology
Specific subject area	Isotope analyses
Type of data	Table Figure Graph
How data were acquired	Fourier transform infrared spectroscopy (FTIR); VG OPTIMA Isocarb isotope ratio mass spectrometer (IRMS); thermal ionization mass spectrometer (TIMS)
Data format	Raw Analysed
Parameters for data collection	Permanent second molars (M2) were selected (n=20) from an equal number of male (n=10) and female (n=10) individuals, providing a sex balanced sample. Permanent second molars were chosen as a control for age, based on crown development of the permanent second molar being complete by ca. 7 to 8 years of age.
Description of data collection	Utilizing Fourier transform infrared spectroscopy (FTIR), crystallinity index (CI) values for a subsample of individuals (n=5) were calculated to assess apatite preservation at Velia [4]. Ground enamel samples were then prepared for δ^13^C_carbonate_ and δ^18^O_carbonate_ VPDB [5] and ^87^Sr/^86^Sr [6] mass spectrometric analyses in reference to NIST SRM-987 = 0.710260 ± 0.000010 [7,8], and NBS-19 where δ^13^C VPDB = +1.95‰ and δ^18^O VPDB = −2.20‰ [7,9]. Resultant δ^18^O_carbonate_ VPDB values were converted to δ^18^O_carbonate_ VSMOW [10], δ^18^O_dw_[11], and δ^18^O_phosphate_ VSMOW [12] to facilitate comparability.
Data source location	City/Town/Region: Velia, Campania Country: Italy samples/data: Specimens were analysed at McMaster University in the School of Geography and Earth Sciences (^87^Sr/^86^Sr) and McMaster Research for Stable Isotopologues (MRSI) (δ^18^O and δ^13^C).
Data accessibility	Repository name: IsoArcH [13] Data identification number: https://doi.isoarch.eu/doi/2021.002
Related research article	R.J. Stark, M.V. Emery, H. Schwarcz, A. Sperduti, L. Bondioli, O.E. Craig, T. Prowse, Imperial Roman mobility and migration at Velia (1^st^ to 2^nd^ c. CE) in southern Italy, J. Arch. Sci.: Reports 30 (2020) 102217. 10.1016/j.jasrep.2020.102217.

## Value of the Data

• These data reflect the first ^87^Sr/^86^Sr, δ^13^C_carbonate_, and δ^18^O_carbonate_ values derived from Imperial Roman bioarchaeological contexts at Velia and more broadly Campania, Italy.
• These data will be useful to bioarchaeological researchers investigating questions of diet, mobility, and migration, particularly for the Imperial Roman era and for contexts in southwestern Italy.
• These data can be utilized for comparisons to ^87^Sr/^86^Sr, δ^13^C_carbonate_, and δ^18^O_carbonate_ values from other sites as well as for future research at and around Velia. The data presented may be utilized for subsequent studies of the same individuals through integration into skeletal biological, palaeodemographic, palaeogenetic, palaeopathological and/or additional isotopic analyses (e.g. S, Pb).

## 1 Data Description

**Fig. 1:**
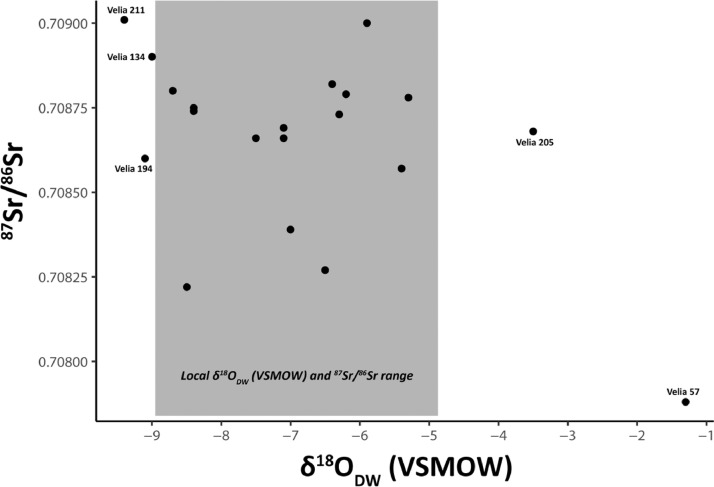
Scatter plot of ^87^Sr/^86^Sr and δ^18^O_dw_ VSMOW values for individuals sampled from Velia (n=20) with expected local ranges shown in grey, as defined by the 2σ range of nine archaeofaunal pig teeth for ^87^Sr/^86^Sr [1,14] and meteorological precipitation data [2] considered in conjunction with the local range at Portus [3] for δ^18^O_dw_ (after Supplementary Fig. 1 in [1]).

**Fig. 2:**
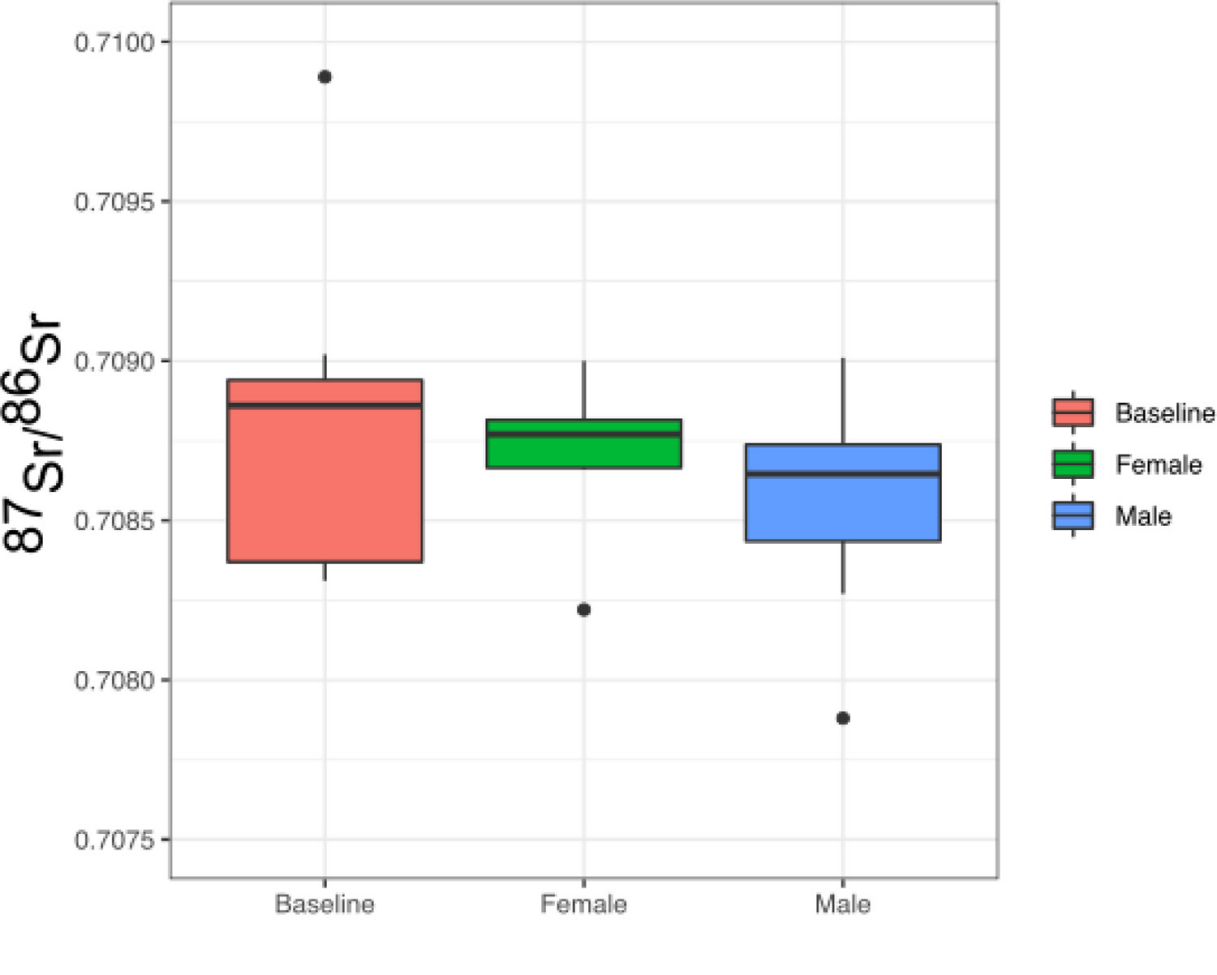
Boxplot showing variation between Male (*n* = 10) and Female (*n* = 10) ^87^Sr/^86^Sr values in relation to expected local bioavailable ^87^Sr/^86^Sr values for the area around Velia derived from pigs teeth (*n* = 9).

**Fig. 3:**
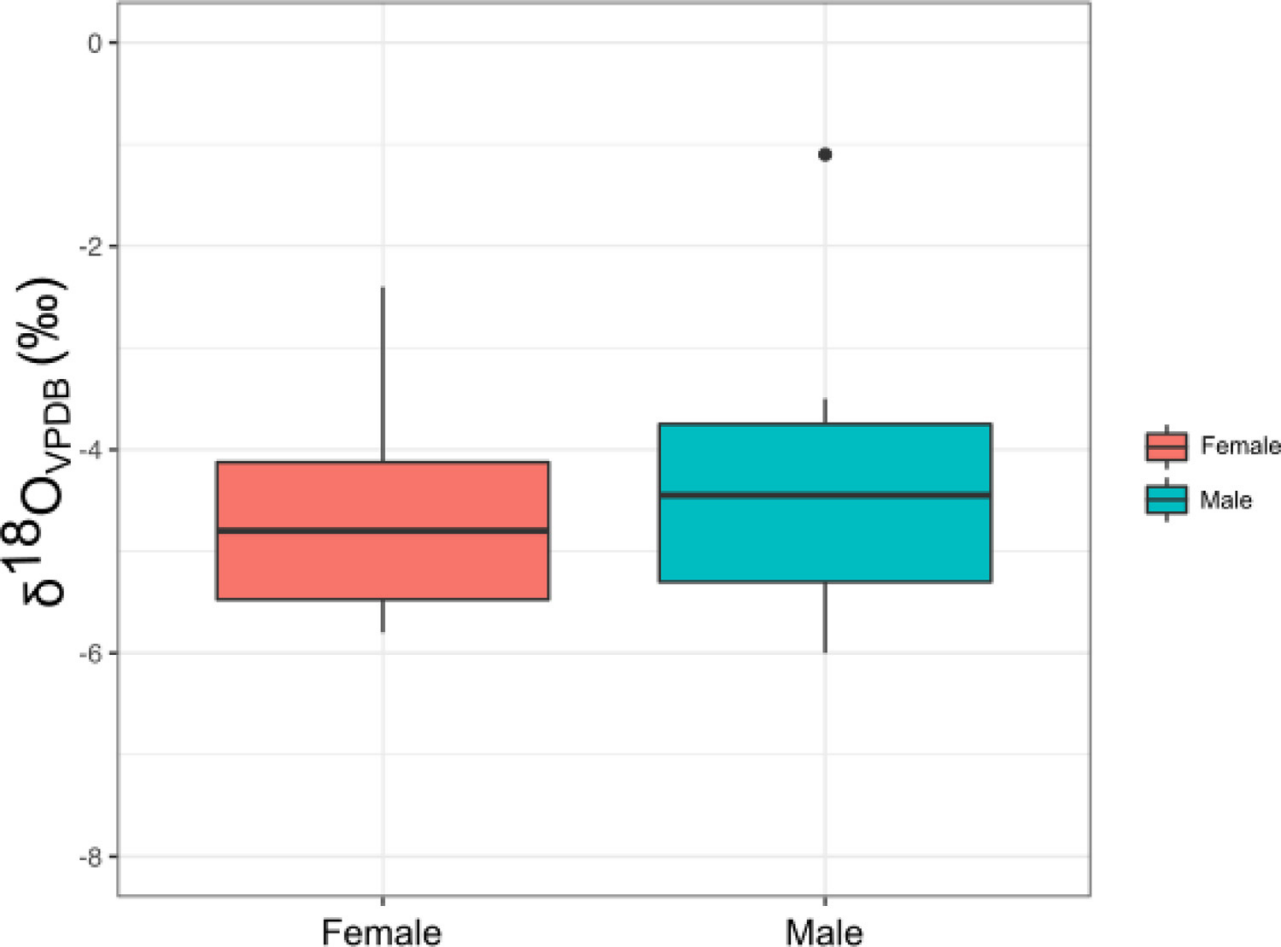
Boxplot showing variation between Male (*n* = 10) and Female (n=10) δ^18^O_c_ VPDB values.

The data comprise crystallinity index (CI) values of five individuals from Velia, strontium (^87^Sr/^86^Sr), carbon carbonate (δ^13^C_carbonate_ VPDB), and oxygen carbonate (δ^18^O_carbonate_ VPDB) values using delta notation (δ) in per mil increments (‰). The presented values were derived from the permanent second molars (M2) of twenty individuals (10 male and 10 female) dated to Imperial Roman (ca.  1^st^ to 2^nd^ c. CE) contexts at the site of Velia located in the Cilento of Lucania, modern day Campania, Italy. Table 1 presents dental enamel crystallinity index (CI) values for five randomly selected individuals (two males and three females). Given the uniform environmental contexts of deposition at Velia, the five sampled individuals were utilized as a gauge of apatite preservation for the broader site environs. Table 2 presents sample data by individual, providing sex and age of the individuals analysed, original δ^18^O_carbonate_ VPDB values, converted δ^18^O_carbonate_ VSMOW values, converted δ^18^O_phosphate_ VSMOW values, expected δ^18^O values in meteoric precipitation (δ^18^O_dw_), δ^13^C_carbonate_ values, and ^87^Sr/^86^Sr values with associated errors. The δ^18^O and ^87^Sr/^86^Sr data presented in Table 1 were imported into ‘R’ (https://www.r-project.org/) in CSV file format to facilitate statistical analyses and generation of a series of graphical representations of the data characteristics. A scatterplot of the ^87^Sr/^86^Sr and δ^18^O_dw_ VSMOW data with associated expected local ranges for ^87^Sr/^86^Sr and δ^18^O_dw_ VSMOW is presented in Fig. 1. Expected local bioavailable ^87^Sr/^86^Sr for Velia was established based on the 2σ range of values from nine archaeofaunal pig teeth [1], while the expected local range for δ^18^O was determined based on meteorological precipitation data [2] considered in conjunction with the local range for dental values at Portus [3], which falls within the same isopleth as Velia. To consider the variation between the expected local bioavailable baseline for ^87^Sr/^86^Sr at Velia, as derived from nine pig teeth, a boxplot presenting the expected local ^87^Sr/^86^Sr baseline, male (n = 10 individuals), and female (n = 10 individuals) ^87^Sr/^86^Sr values is presented in Fig. 2. Similarly, a boxplot comparing the δ^18^O_carbonate_ VPDB of male (n = 10) and female (n = 10) individuals analysed from Velia is presented in Fig. 3.

**Table 1:** Crystallinity index (CI) values of five sampled individuals from Velia.

Individual	Sex	A_565_	A_605_	A_595_	CI Value
Velia 134	F	0.59	0.55	0.30	3.8
Velia 146	M	0.68	0.62	0.36	3.6
Velia 194	M	0.36	0.33	0.19	3.6
Velia 205	F	0.23	0.21	0.13	3.3
Velia 214	F	0.24	0.21	0.12	3.7

**Table 2:** Sex and age of individuals sampled from Velia, raw δ^13^C_carbonate_ VPDB, δ^18^O_carbonate_ VPDB, and ^87^Sr/^86^Sr values, with converted δ^18^O_carbonate_ VSMOW, δ^18^O_phosphate_ VSMOW, and δ^18^O_dw_ values (modified from [1]).

		Age	δ^13^C_carbonate_	δ^18^O_carbonate_	δ^18^O_Carbonate_	δ^18^O_Phosphate_	δ^18^O_dw_		^87^Sr/^86^Sr
Site	Sex	(Years)	VPDB (‰)	VPDB (‰)	VSMOW (‰)	VSMOW (‰)	(‰)	^87^Sr/^86^Sr	Error ±
Velia 57	M	30-35	−12.4	−1.1	29.8	20.7	−1.3	0.70788	0.00002
Velia 82	F	50+	−12.3	−4.1	26.7	17.7	−6.2	0.70879	0.00015
Velia 117	F	20-30	−13.0	−5.6	25.1	16.1	−8.7	0.70880	0.00002
Velia 134	F	20-30	−13.3	−5.8	24.9	15.9	−9.0	0.70890	0.00002
Velia 139	M	30-40	−13.0	−4.6	26.2	17.1	−7.0	0.70839	0.00015
Velia 146	M	43-55	−12.9	−4.3	26.5	17.5	−6.5	0.70827	0.00002
Velia 160	F	30-40	−11.5	−4.9	25.9	16.8	−7.5	0.70866	0.00002
Velia 169	M	30-40	−12.9	−5.5	25.3	16.3	−8.4	0.70874	0.00015
Velia 174	M	40-50	−12.9	−4.7	26.1	17.1	−7.1	0.70869	0.00002
Velia 181	F	50+	−13.1	−4.7	26.1	17.1	−7.1	0.70866	0.00002
Velia 182	M	25-30	−13.3	−4.2	26.6	17.6	−6.3	0.70873	0.00005
Velia 186	M	20-24	−12.8	−3.6	27.2	18.1	−5.4	0.70857	0.00002
Velia 194	M	30-40	−12.4	−5.8	24.9	15.9	−9.1	0.70860	0.00002
Velia 205	F	30-40	−13.6	−2.4	28.4	19.3	−3.5	0.70868	0.00025
Velia 211	M	30-35	−12.8	−6.0	24.7	15.7	−9.4	0.70901	0.00002
Velia 214	F	25-35	−13.3	−5.5	25.3	16.3	−8.5	0.70822	0.00002
Velia 222	M	30-40	−12.7	−3.5	27.3	18.2	−5.3	0.70878	0.00025
Velia 223	F	40-45	−12.9	−5.4	25.3	16.3	−8.4	0.70875	0.00003
Velia 270	F	40-50	−13.2	−3.9	26.9	17.8	−5.9	0.70900	0.00005
Velia 283	F	50+	−13.1	−4.2	26.6	17.5	−6.4	0.70882	0.00002

## 2 Experimental Design, Materials and Methods

### 2.1 Sampling approach

The rationale for collecting the data presented herein was to compare ^87^Sr/^86^Sr and δ^18^O_c_ values preserved in human dental enamel to expected local ^87^Sr/^86^Sr and δ^18^O_dw_ values for the region around Velia, to assess for possible instances of mobility to this Imperial Roman (ca. 1st to 2nd c. CE) secondary port settlement located in the Cilento of Lucania. Permanent second molars (M2) from twenty adult individuals interred at Velia were collected in 2012 and 2013 from the Museo delle Civiltà (formerly the Museo Nazionale Preistorico Etnografico “L. Pigorini”) in Rome, Italy where the human skeletal remains from Velia are curated. All sampled individuals were recovered from inhumation burials. A sex balanced sample of 10 males and 10 females was chosen to provide equal representation for comparison of isotope values. Following collection in Rome, all M2 samples were prepared for isotopic analyses at McMaster University, in Hamilton, Ontario, Canada.

### 2.2 Dental enamel sample pre-treatment

All of the human second molars (M2) subjected to isotopic analyses were initially manually brushed to remove any adhering debris before being submersed in distilled water (dH_2_O) and ultrasonicated for a period of 10 min. Ultrasonication was repeated three times changing the water for each rinse, after which the teeth were dried in a drying oven at 60 °C before being drilled to remove enamel for sampling.

### 2.3 FTIR and CI analyses

Testing for diagenetic alteration of apatite quality for the Velia samples was completed through subsampling of five randomly selected individuals (Velia 134, 146, 194, 205, 214) at the McMaster Combustion Analysis and Optical Spectroscopy Facility using Fourier transform infrared spectroscopy (FTIR). FTIR spectra were utilized to calculate crystallinity (CI) index according to CI = (A_565_ + A_605_)/A_595_, where A_x_ is the absorbance at wave number x, assuming a straight line baseline between 750 and 450 cm^−1^ [4]. Samples for FTIR analysis were first cleaned before the enamel was ground into a fine powder and passed through a #200 mesh sieve. Each ground enamel sample was combined with dry potassium bromide (KBr) and ground before being compressed into pellets at 10,000 psi. The compressed pellets were then analysed using a Nicolet 6700 dry nitrogen purged FTIR, room temperature DTGS detector with extended KBr beam splitter, resolution 4 cm^−1^ (wavenumber) at 32 scans. CI values for all five individuals were ≤3.8, indicating sufficient apatite preservation and absence of diagenetic alteration (Table 1).

### 2.4 Sample preparation for δ^13^C and δ^18^O VPDB analysis

From each molar sampled, ≥10 mg of powdered dental enamel was removed using a diamond tipped drill bit in a hand-held electric Dremel drill. After each use the drill bit was soaked in 0.25M hydrochloric acid (HCl) for ca. 10 min to avoid cross contamination and then rinsed in distilled water (dH_2_O). After weighing, enamel powder was collected in 1.5 ml plastic centrifuge microtubes.

Powdered enamel samples were treated with 0.04 ml of 2.5% bleach solution (NaClO) per mg of sample after which they were agitated and allowed to react for a period of up to 24 h. Following this reaction, samples were centrifuged and rinsed with de-ionized water five times, centrifuging after each rinse. Each sample next had 0.04 ml of 1M acetic acid acetate buffer (CH_3_COOH) per mg of sample added to remove potential diagenetic secondary carbonates. Samples were agitated and allowed to react for a period of up to 24 h. Samples were then centrifuged and rinsed five times with de-ionized water, centrifuging after each rinse. After the fifth rinse samples were centrifuged and the remaining water removed before the teeth were dried in a drying oven at 60 °C [5].

Once the samples were dry, 2 mg of enamel powder was weighed into stainless steel cups. Each sample was reacted with 100% phosphoric acid at 90 °C in an autocarb analyser to produce CO_2_ gas, which was analysed on a VG OPTIMA Isocarb isotope ratio mass spectrometer (IRMS) at the McMaster Research for Stable Isotopologues (MRSI) laboratory to measure δ^18^O and δ^13^C values. For each carousel containing 14 samples one sample was run in duplicate to test for accuracy and reproducibility, which is to say 13 samples and 1 duplicate were run with each carousel. Returned δ^18^O and δ^13^C values are presented using delta notation (δ) defined as,

$$\delta^{18}O_{x}=\left\{\left[{\left({}^{18}O{/}^{16}O\right)}_{x}/{\left({}^{18}O{/}^{16}O\right)}_{std}\right]-1\right\}\times 1000$$

where, x = sample and std = standard, presented in per mil (‰) increments in reference to the Vienna Pee Dee Belemnite (VPDB) standard where NBS-19 δ^18^O VPDB = −2.2‰ and δ^13^C VPDB = +1.95‰ [7]; precision of analysis is ±0.2‰. The data have been corrected here on the premise that the CO_2_–CO_3_ offset is the same as that for acid reaction of calcite (CaCO_3_) at the same temperature; this was controlled by periodic reaction of a standard calcite (NBS-19) at the same temperature. The returned δ^18^O_carbonate_ VPDB values were then converted to Vienna Standard Mean Ocean Water (VSMOW) values according to δ^18^O_carbonate (VSMOW)_ = 1.0309 × δ^18^O_carbonate  (VPDB)_ + 30.91 [10], and then subsequently to approximated meteoric water values (δ^18^O_dw_) according to, δ^18^O_dw_ = 1.590 × δ^18^O_carbonate (VSMOW)_-48.634 [11] to allow for comparison to documented δ^18^O_dw_ in global meteoric precipitation. For transparency in data presentation and to facilitate broader comparative value of these data, δ^18^O_carbonate (VSMOW)_ values were additionally converted to δ^18^O_phosphate (VSMOW)_ values according to δ^18^O_phosphate (VSMOW)_ = 0.98 × δ^18^O_carbonate (VSMOW)_ – 8.5 [12].

### 2.5 Sample preparation for ^87^Sr/^86^Sr

From each molar sampled, ≥60 mg of powdered dental enamel was removed using a diamond tipped drill bit in a hand-held electric Dremel drill. After each use the drill bit was soaked in 0.25M HCl for ca. 10 min to avoid cross contamination and then rinsed in distilled water (dH_2_O). After weighing, enamel powder was collected in 1.5 ml plastic centrifuge microtubes.

Strontium extraction followed the protocol of the thermal ionization mass spectrometry (TIMS) laboratory directed by Dr. Alan Dickin in the School of Geography and Earth Sciences at McMaster University. Enamel was initially dissolved in 1.2 ml of 2.5 M HCl. Following full dissolution of the enamel, samples were centrifuged for 10 min. Cation exchange was employed to complete the strontium separation. Cation exchange columns were calibrated by employing a test “spiked” sample allowing for the stage of Sr collection to be assessed. In order to cleanse the cation exchange columns before use, 10 ml of deionized water was introduced after which a wash of 60 ml of 6 M HCl was introduced, followed by 10 ml of deionized water, and then finally 5 ml of 2.5 M HCl.

Dissolved enamel solution for each individual was introduced to the exchange columns in 1 ml portions and was washed into the column using 1 ml of 2.5 ml HCl, after which a wash of 3 ml of 2.5 M HCl was introduced. Waste sample matrix was eluted using 20 ml of 2.5 M HCl. After the 20 ml elution, 6 ml of 2.5 M HCl was introduced to the columns to collect the strontium. Strontium was collected in 4 ml intervals into Teflon beakers. Once the strontium phase was collected samples were placed under a heat lamp to dry to a solid state. Once dry, each sample was loaded onto a pre-treated single tantalum filament in dilute phosphoric acid, after which the samples were loaded in sequence into a vacuum system [6].

^87^Sr/^86^Sr values of all samples were determined by dynamic multi-collection using a thermal ionization mass spectrometer (TIMS) in the School of Geography and Earth Sciences at McMaster University. Resultant strontium values were fractionation normalized to ^88^Sr/^86^Sr =  .1194, with an average ^87^Sr/^86^Sr = 0.71026±18 (1σ) for the NIST 987 Sr standard and internal precision (within-run precision) of ±0.0012–0.0018% (1σ) standard error based on 150 dynamic cycles.

### 2.6 Data Analysis

Of the 20 samples analysed for ^87^Sr/^86^Sr and δ^18^O, 5/20 (25%) fall outside of the expected local δ^18^O_dw_ range: Velia 57, 134, 194, 205, and 211; none of the individuals sampled fall outside of the expected local bioavailable ^87^Sr/^86^Sr range (Fig. 1). Of the five individuals outside of the expected local δ^18^O_dw_ range, 2/5 (40%) are female (Velia 134 and 205) and 3/5 (60%) are male (Velia 57, 194, and 211). In terms of δ^18^O_c_ (VPDB) values, the interquartile range for both the male and female individuals sampled are overall similar, with males having a slightly less negative median value than females (Fig. 3). The same is not true of ^87^Sr/^86^Sr values, where a much narrower interquartile range of values is evident for the female individuals sampled than the males (Fig. 2). The interquartile ranges for both male and female ^87^Sr/^86^Sr values, however, fall within the interquartile range for the expected local bioavailable ^87^Sr/^86^Sr baseline for the area around Velia as established from pig dental enamel [1,14]. The δ^13^C values of the 20 individuals analysed fall within a relatively narrow range, spanning from −13.6‰ (Velia 205) to −11.5‰ (Velia 160). Considering these δ^13^C values further, the majority of individuals (18/20, 90%) fall between −13.3‰ and −12.3‰, increasingly suggesting overall similarities in whole diet among the individuals sampled. The δ^18^O, δ^13^C, and ^87^Sr/^86^Sr data presented herein indicate that most of the individuals sampled from Velia, both male and female, were likely local to the area around Velia, or similar proximate geological areas.

## Ethics Statement

All research was conducted using bioarchaeological skeletal materials in compliance with the McMaster Research Ethics Board (MREB) of McMaster University. No data were collected from modern human populations or individuals with known relatives.

## CRediT authorship contribution statement

**Robert J. Stark:** Conceptualization, Project administration, Methodology, Validation, Formal analysis, Investigation, Visualization, Writing – original draft, Funding acquisition. **Matthew V. Emery:** Conceptualization, Visualization, Writing – review & editing, Formal analysis. **Henry Schwarcz:** Conceptualization, Writing – review & editing. **Alessandra Sperduti:** Resources, Writing – review & editing. **Luca Bondioli:** Resources, Writing – review & editing. **Oliver E. Craig:** Resources, Writing – review & editing. **Tracy L. Prowse:** Supervision, Funding acquisition, Methodology, Writing – review & editing.

## Declaration of Competing Interest

The authors declare that they have no known competing financial interests or personal relationships which have or could be perceived to have influenced the work reported in this article.
